# Are Virtues, Vices and Habits Hereditary?

**Published:** 1898-06

**Authors:** T. J. Happel

**Affiliations:** Trenton, Tenn.; President Tri-State Medical Association (Miss., Ark. and Tenn.); Ex-President Tennessee State Medical State Society; Ex-President West Tennessee Medical and Surgical Association; Ex-President Gibson County Medicsl Society


### ARE VIRTUES, VICES AND HABITS HEREDITARY?

By T. J. HAPPEL, M.D., TRENTON, TENN.

President Tri-State Medical Association (Miss., Ark. and Tenn.); Ex-President Tennessee State Medical State Society; Ex-President West Tennessee Medical and Surgical Association; Ex-President Gibson County Medicsl Society.

The subject assigned me by your secretary opens up the whole broad field of heredity.

In general terms it is my intention to discuss this question, without giving attention to any of the minute details, whether with regard to the anatomic structure of the nervous system, or any other pathologic question connected with it. Of my own knowledge, I could not trace changes in any of the organs in "degenerates," so called; hence, to write upon that branch of the subject would require a book article, which, on occasions of this kind, does not suit my taste, nor would it be profitable for us to discuss. I purpose taking up and presenting matters of the every-day life of the physician.

The question of the transmission of physical peculiarities from parent to child needs no time devoted to it. All of us have seen children whom we could recognize from their family likeness. Frequently the son would be mistaken for the father, or the daughter for the mother, but for that difference in appearance due to the disparity in their ages. Breeders of fine stock all have the form and make-up of the sires and dam constantly before them in estimating the value of the expected offspring.

In my own experience, I have lately been in professional relationship to a family in which every child, immediately upon its coming into the world, is examined as to the existence of harelip. Through generations this peculiarity has been handed down. In other families we have found six-fingered children; in others, a certain "mother's mark," or mole, as a family characteristic. In other families, we find a marked tendency to strabismus, and in others, to some form of talipes. Thus I might continue to enumerate physical peculiarities handed down from one generation to another. That some exciting cause may produce a mark or departure from the normal in the physical condition of the offspring, needs no argument. It has been the experience of many physicians in this meeting to have had to answer, as soon as the child has been separated from its mother and brought to view, and at other times, as soon as it has been expelled from the uterus, as to the condition of the child, "whether marked or not." And when the reason for the question is asked, they have been told that the woman, when pregnant, had been frightened by a snake, a frog, or some unsightly object.

Jacob, serving Laban for his wives, became rich, when by an agreement with his father-in-law he fell heir to all the "ring-streaked, spotted, and speckled young," he having, through maternal impressions, caused a wonderful number of that kind of progeny. In the Holy Writ we are told that the sins of the fathers are visited upon the children even of the third and fourth generations. Other matter might be cited along this line, but suffice it to say that some men's sins do go before them unto judgment, and others follow after them.

I do not desire to be understood as taking the position that a disease is, as a rule, handed down through heredity to the children of the first generation. We have only to look around us to disprove this position.

A father and mother lived to be more than fourscore years of age, enjoying a reasonable degree of health and strength, but of their children, two sons and one daughter died of tueberculosis, and the end is not yet.

The transmission of syphilis by heredity is not questioned in this age, but a syphilitic father may beget children who, so far as we can judge, show no trace of the disease, but the offspring of those children may, and frequently do, give evidence of some dyscrasia, whether syphilitic or tubercular. A generation in which the rule had been perfect health passed away before the vice of the parent was exhibited.

When we contend that virtues and vices are transmitted by heredity, we do not take the position that, as in the transmission of physical marks, like produces like, but that there is begotten in the offspring of the degenerate a certain peculiarity of nervous development that may, at any moment, show some abnormal state. An epileptic may not always beget an epileptic child, but in some cases other brain infirmities may be shown. An imbecile may result from the marriage of epileptics. An idiotic child was the result of the marriage of two beings of a very low order of intellect whom I knew. A child begotten by a father whilst in a drunken debauch may, nay frequently proves to be a degenerate.

A friend reports to me the case of two beautiful daughters begotten by a father who was never free from alcoholic influence, both of whom are epileptics. So far as the family history is known, there had never been a case of epilepsy in either paternal or maternal branch. Children born of such parentage may become addicted to the use of alcohol, or may become morphine habitues, or maniacs. The medical and surgical history of the war of the rebellion reports that in a village in one of the New England States, as a result of intermarriages—many of the population being epileptics and imbeciles—almost the whole population showed abnormalities of various kinds to such an extent that not one in the community was fit for military service.

In my own experience as a practitioner of medicine for twenty-five years, I have known five members of one family to die of cancer; two of the breast, one of the brain, one of the liver, and one of a colloid cancer springing from the neck of the bladder. In this family the dyscrasia can be traced back through generations, so that every member of it now living looks with horror upon any sore or localized pain wherever found, expecting a development of the dreaded scourge.

In our every-day life we can see the drunkard's children following closely in the footsteps of the father, unless the mother had sufficient individuality to impress the children with some of her own better qualities. My experience, however, is that the sons are more apt to be impressed by the father's vices, the daughters by the mother's, but this is not laid down as a rule.

In an article presented by me some years ago to the Tennessee State Medical Society upon the question of morphinism, I gave the history of one mother, a morphine habitue, who gave birth to four children, three of whom died within one week, with every evidence of cyanosis, due to non-closure of the foramen ovale (caused by the use of morphine on the part of the mother), and showing nervous symptoms that could be allayed only by the use of opiates. The fourth child born to this mother lived about six months, demanding during the first few weeks of its existence large doses of paregoric to quiet it and give it sleep. It was at all times a poorly nourished child, showing dyscrasias of various kinds during its short life.

Another mother was reported as having given birth to three children under similar circumstances, two of whom died in exactly the same condition as did the first ones mentioned in the preceding case, whilst the third child now lives, a delicate, puny, nervous child. Another mother gave birth to two children, one being still-born, the other living, aged now, three years. This child required large doses of opiates to carry it through its first month.

Another mother, using morphine freely, aborted twice in the early months of pregnancy. She has now one grown son, who has for a long time been addicted to the use of morphine. To a father who used morphine in enormous doses a son was born, who, at the age of seven years, is just able to talk intelligibly. To a father who was addicted to the use of both morphine and whisky, two sons and two daughters were born. Both of the sons became sots and morphine eaters, and in early manhood one died; the other is a mental and physical wreck. The girls, so for as I know, show no dyscrasias of any kind. The mother had never used drugs.

From another source, I find that a child was born to a morphine-using mother, which child was nervous and fretful from its birth, and after various means had been resorted to quiet it, was given morphine. From that moment the child was never at rest except when under the influence of the drug. A mother who, whilst pregnant, craved alchohol, and used liquor frequently during the whole nine months, gave birth to twin boys. Growing up to manhood, nervous and irritable, both died confirmed sots.

It might, however, be argued that in some of the cases cited above, the vice developed, not from heredity, but as a result of being brought habitually in contact with drunken fathers or morphine-eating mothers, or vice versa, but case after case can be cited where children born to sots or opium habitues had been removed to entirely new environments, where they knew nothing of their parents or their habits, and yet the same sad story has been repeated in their cases. I do not wish to be understood as contending that this rule holds good in all cases in these two vices, but, as in tubercular subjects, a whole generation may escape. Again, in my own work, I have recently seen a father, a slave to alchohol, die of pneumonia, and within less than two months of his death, a son, who had suffered, six months before, from an attack of apoplectic congestion, died of softening of the brain. The son had never used alcohol in any form, but had never been mentally bright, though a steady worker. The probabilities are that the son was begotten during a drunken debauch, and the sins of the father were thus transmitted to the son.

Mental peculiarities, virtues as well as vices, can be traced through several generations. In some families the trend is toward the ministry; in another, to the law; in another, to medicine; and in another, to politics.

From the *Herald of Health* I gather the following: Demme studied ten families of drinkers and the same number of temperate persons. The ten families of drinkers produced fifty-seven children. Of these twenty-five died within the first few months of their life; six were idiots; five showed abnormalities of growth; five were affected with epilepsy; five with inborn diseases, and one with chorea. Thus, then, of the fifty-seven children, only ten, or 17.5 per cent., born to drinkers, were normal in all respects. The other group of sober families had sixty-one children. Five died in the first week; four had curable diseases of the nervous system, and only two presented inborn defects. The remaining fifty, or 81.9 per cent., were normal in their physical and mental development. Thus it is shown that the prevailing mortality among the children of drunkards is fearful, and that the survivors represent a progeny afflicted with unsoundness of mind, idiocy, epilepsy, and various other nervous disturbances, whilst only a small per cent. of them grow up to be useful members of society.

This paper thus far has treated of the more important feature of heredity, namely, the transmission of vices, but the converse of the proposition is also true. Virtues also descend from parent to child, not always as the same virtue, but the sound body and sound mind furnesh fruitful soil for the germination of the good seed sown by the sower. In every community families can be traced through generations without revealing vices in the children, and when in the course of time in some branch of the family we find a departure from the virtues of the other branches, this can easily be shown to be due to heredity from the other side of the family tree. *Memphis Medical Monthly.*

